# Tai chi for overweight/obese adolescent and young women with polycystic ovary syndrome: study protocol for a randomized controlled trial

**DOI:** 10.1186/s13063-018-2893-z

**Published:** 2018-09-20

**Authors:** Yan Li, Changle Peng, Guangying Cao, Wei Li, Lihui Hou

**Affiliations:** 1 First Affiliated Hospital, Heilongjiang University of Chinese Medicine, 26 Heping Road, Harbin, 150040 China; 2 Qiqihar First Hospital, 30 Longsha Garden Street, Qiqihar, 161005 China; 3 Heilongjiang University of Chinese Medicine, 24 Heping Road, Harbin, 150040 China

**Keywords:** Polycystic ovary syndrome, Tai chi, Complementary and alternative medicine

## Abstract

**Background:**

Tai Chi is a moderately intense exercise that dates back to ancient China. It has been reported that Tai Chi not only has beneficial effects on metabolic disorders, such as diabetes, cardiovascular diseases and obesity, but also has favorable effects on psychological well-being. Since these conditions are quite closely associated with polycystic ovary syndrome (PCOS), we hypothesis that Tai Chi could be a potential treatment option for PCOS patients. We aim to determine the feasibility and effectiveness of Tai Chi on overweight/obese adolescent and young women with PCOS.

**Methods:**

A total of 50 patients will be randomized into two arms: (1) Tai Chi or (2) self-monitored exercise. Both groups will exercise for 3 months. The primary hypothesis is that Tai Chi results in a significantly lower Body Mass Index (BMI) than self-monitored exercise. The study was approved by the Ethics Committee of the First Affiliated Hospital of Heilongjiang University of Chinese Medicine.

**Discussion:**

This is the first study to determine the feasibility and effectiveness of Tai Chi in treating overweight/obese adolescent and young women with PCOS. The trial will provide evidence to assess the feasibility of a future multicenter, randomized controlled trial.

**Trial registration:**

ClinicalTrials.gov, ID: NCT02608554. Registered on 17 November 2015.

**Electronic supplementary material:**

The online version of this article (10.1186/s13063-018-2893-z) contains supplementary material, which is available to authorized users.

## Background

Polycystic ovary syndrome (PCOS) is the most common endocrine disorder and affects 5–20% of reproductive-age women [1]. It is a complex syndrome characterized by reproductive and metabolic implications including amenorrhea /oligomenorrhea, hyperandrogenism (including hirsutism) or acne and very often by overweight and obesity [2, 3].

Overweight or obesity in adults has been defined by the World Health Organization (WHO) (Body Mass Index (BMI) ≥ 25 kg/m^2^ for overweight, BMI ≥ 30 kg/m^2^ for obesity). In studies on Asian subjects, overweight is considered to be a BMI ≥ 23 kg/m^2^and obesity a BMI ≥ 25 kg/m^2^ [4]. For adolescents, age- and gender-specific percentile distributions for BMI using the Centers for Disease Control and Prevention growth charts were used to identify those who were overweight (85th–95th percentile) or obese (> 95th percentile) [5].

The prevalence of overweight and obesity in women with PCOS is highly variable since it differs in the general population according to age, ethnicity and geographic regions. A meta-analysis reported that the prevalence of obesity (BMI ≥ 30 kg/m^2^) in women with PCOS ranged from 12.5 to 100% with a pooled estimated prevalence of 49% (95% CI 42–55%); the prevalence of overweight (BMI ≥ 25 kg/m^2^) and obesity (BMI ≥ 30 kg/m^2^) ranged from 6 to 100% with a pooled estimated prevalence of 61% (95% CI 54–68%). Also, the prevalence of central obesity was significantly higher in women with PCOS compared with controls [6].

Despite the highly variable reported prevalence of overweight and obesity in women with PCOS, it is confirmed that being obese is significantly associated with worse metabolic and reproductive outcomes measured, except for hirsutism, when compared to normal-weight women with PCOS [6]; whereas women who were overweight but not obese only had no differences in total testosterone (T), hirsutism, total cholesterol and low-density lipoprotein cholesterol (LDL-C) compared to normal-weight women, and no differences in sex-hormone-binding globulin (SHBG), total T and fasting lipids compared to obese women [6]. Central obesity was associated with higher fasting insulin [6]. Also, overweight and obesity is associated with unfavorable in vitro fertilization (IVF)/ intracytoplasmic sperm injection (ICSI) outcomes in PCOS patients treated with a gonadotropin-releasing hormone (GnRH) agonist using the long protocol [7, 8].

Therefore, it is of great importance to be aware that overweight and obese PCOS patients, especially adolescent and young women with PCOS, should also be advised to lose weight. This will not only assist with their immediate symptoms but also will help to prevent the development of diabetes and cardiovascular disease in later life. Lean women with PCOS should be cautioned of the high risk of overweight, obesity and central obesity associated with PCOS, and be encouraged to engage in health behaviors that prevent weight gain.

It is confirmed that even modest weight loss of just 5% body weight in obese women with PCOS can restore regular menstruation and improve response to ovulation induction and fertility treatment [9]. Lifestyle modification, use of metformin, taking hormonal contraceptives (HCs), and bariatric surgery are the main treatment options in obese patients with PCOS recommend by guidelines [10]. Lifestyle modification is the first-line management which could both prevent weight gain and induce weight loss. However, improved engagement and sustainability remain challenges while using. Medications such as metformin, HCs and bariatric surgery have been used with the need for large-scale randomized clinical trials to define their roles and side effects. Novel therapies including inositols [11] and statins [12] have been under investigation lately. Complementary and alternative medicine approaches such as Chinese medicine formulas [13] and acupuncture [14] are also under study. Their efficacy and safety still need to be addressed. More research is needed to assign better therapeutic regimens in overweight and obese PCOS patients and strategies to prevent the development of comorbidities.

Tai Chi is an exercise system followed Chinese Medicine theory which dates back to seventeenth century. It can maintain the harmony between *qi* and the blood, keep *yin* and *yang* in balance and has traditionally been used in various forms for the promotion and maintenance of health and longevity in China [15]. Recently, physiological and psychosocial benefits of Tai Chi on chronic diseases which are very closely related to PCOS, such as obesity, cardiovascular diseases (CVD) and type-2 diabetes [16–18], have been addressed.

A recently published randomized controlled trial of 374 Chinese adults reported that, a 12-week Tai Chi training led to significant loss of 0.50 of body weight and 0.47 of fat mass compared with a control group, which is a similar effect as in a walking group [19]. Another randomized clinical trial consisting of 266 Chinese adults with hypertension reported that a 12-month Tai Chi training could significantly lower BMI compared with a control group (22.25 ± 2.91 vs 23.16 ± 2.94, *p* < 0.001) [16].

To the best of our knowledge, there is no study so far investigating the effect of Tai Chi in women with PCOS. Therefore, the purpose of the present study is to evaluate the effect of Tai Chi in PCOS and the feasibility of executing a large-scale clinical trial.

## Methods

### Study design

**Fig. 1:**
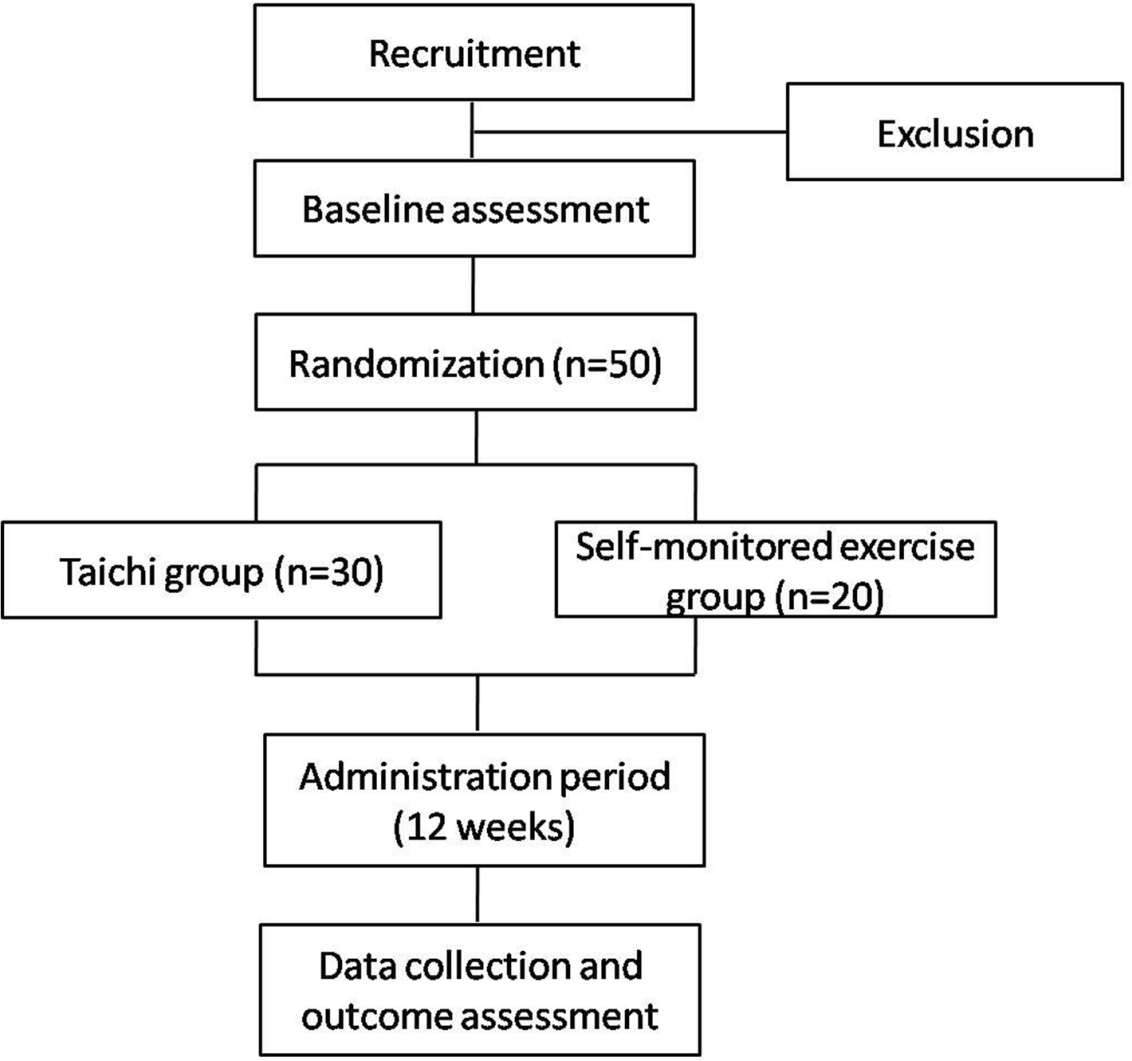
Study design flowchart

**Fig. 2:**
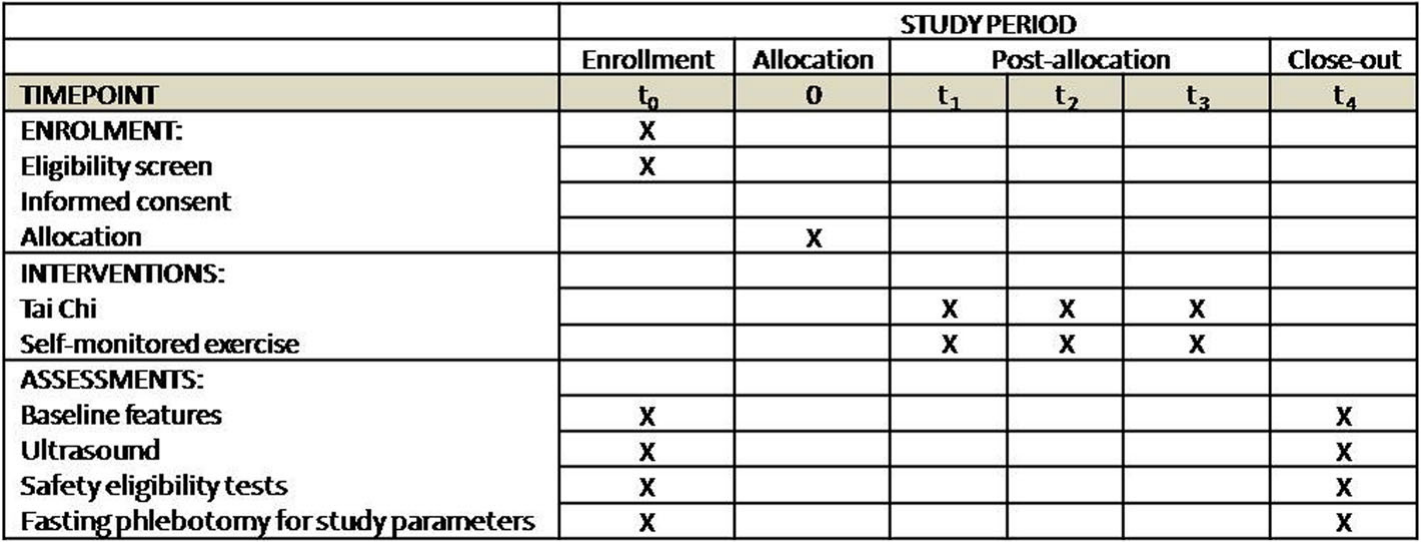
Schedule of enrollment, interventions and assessments (Additional file 1). Safety eligibility includes liver function, renal function and complete blood count (CBC). t1, 1 month after allocation; t2, 2 months after allocation; t3, 3 months after allocation, t4, the beginning of the 4th month after allocation

This present study is a single-blind (assessor), parallel, randomized, pilot feasibility study, which will be carried out to compare Tai Chi with self-monitored exercise in treating PCOS patients. Subjects will be randomly assigned to a Tai Chi group and a self-monitored exercise group with an allocation ratio of 3:2. The study period is 3 months. A flowchart of the study is depicted in Fig. 1. The schedule of trial enrollment, interventions and assessments is provided in Fig. 2 (Additional file 1). This protocol was written following the Standard Protocol Items: Recommendations for Interventional Trials (SPIRIT) Checklist (see Additional file 1).

### Study setting

The present study is conducted in the First Affiliated Hospital, Heilongjiang University of Chinese Medicine. All study visits and Tai Chi interventions will take place at the above mentioned hospital. Self-monitored exercise will be held in a home-based environment.

### Inclusion criteria

- Women aged between 18 and 35 years [19]
- Confirmed diagnosis of PCOS according to the modified Rotterdam criteria and all subjects must have anovulation plus either polycystic ovaries and/or hyperandrogenism
- 2 years after menarche
- BMI equal to or greater than 23 kg/m^2^ [4]
- With no desire to have children within 6 months

PCOS is defined by the modified Rotterdam criteria as: oligomenorrhea or amenorrhea, together with presence of ≥ 12 antral follicles (≤ 9 mm) and/or ovarian volume > 10 mL on transvaginal scanning, and/or clinical/biochemical hyperandrogenism. Oligomenorrhea is defined as an intermenstrual interval > 35 days and less than eight menstrual bleeds in the past year. Amenorrhea is defined as an intermenstrual interval > 90 days. Clinical hyperandrogenism in mainland China is defined with a Ferriman-Gallwey (FG) score ≥ 5 [20].

### Exclusion criteria

- Administration of other medications known to affect reproductive function or metabolism within the past 3 months, including oral contraceptives, gonadotropin-releasing hormone (GnRH) agonists and antagonists, anti-androgens, gonadotropins, anti-obesity drugs, Chinese herbal medicines, anti-diabetic drugs such as metformin and thiazolidinediones, somatostatin, diazoxide and calcium-channel blockers
- Patients with other endocrine disorder including 21-hydroxylase deficiency, hyperprolactinemia, uncorrected thyroid disease, suspected Cushing’s syndrome
- Patients with known severe organ dysfunction or mental illness

### Interventions

#### Tai Chi

Subjects in the Tai Chi group will practice in the gymnasium of Heilongjiang University of Chinese Medicine. The program of Tai Chi training consisted of a 60-min exercise session, three times per week for 12 weeks, based on their original level of physical activity. Each session comprised 40 min of Tai Chi training plus a 10-min warm-up and cool-down. Tai Chi training was instructed by the same experienced Tai Chi instructors who were qualified in teaching. The 24 forms of simplified Tai Chi recommended as a popular health-benefitting sport by the General Administration of Sport of China was applied. Practice attendance will be monitored and adherence to practice will be recorded.

#### Self-monitored exercise

The subjects of the self-monitored exercise group will be asked to conduct extra exercise besides their routine exercise. The self-monitored exercise consists of brisk walking, cycling, jogging, or any other aerobic exercise for 60 min, three times per week for 12 weeks [21]. Adherence to exercise will be tracked via self-report logs, which will be obtained at the weekly frequency during exercise.

## Outcome measurements

### Primary outcome

The primary outcome measurement is the BMI change from baseline. BMI will be calculated using weight (kg)/height (m^2^).

### Secondary outcomes

- Oral glucose tolerance test (OGTT): serum for glucose and insulin levels will be determined
- Hormonal profile including: testosterone (T), androstadiendione (AND), SHBG, dehydroepiandrosterone sulfate (DHEAS), follicle-stimulating hormone (FSH), luteinizing hormone(LH),and estradiol (E2)
- Fasting-lipid metabolic profile: cholesterol, triglycerides (TG), cholesterol (CHOL), high-density lipoprotein cholesterol (HDL-C) and low-density lipoprotein cholesterol (LDL-C)
- Weight, waist/hip circumference, blood pressure, FG score and presence of acne before and after treatment
- Feasibility outcomes including participant recruitment rates, retention rates, treatment fidelity monitored through attendance records
- Adverse events

### Sample size calculation

This study is an exploratory study of the evaluation of the effectiveness of Tai Chi for PCOS subjects and the feasibility of a large clinical trial; therefore, the minimum sample size necessary to evaluate the effectiveness was used [22].

For such a trial designed with 90% power and two-sided 5% significance,

the sample size was set to 50 patients with an allocation ratio of 3:2.

### Randomization and allocation concealment

After the baseline evaluation, 50 subjects will be allocated randomly into one of the two groups in a ratio of 3:2. The identification code and random number, which are unique for each subject, were generated using SAS 9.2 by an independent agency (TCM Translational Medicine Research Centre, First Affiliated Hospital, Heilongjiang University of Chinese Medicine). These assignments were put into sealed, opaque envelopes. The envelopes will only be opened after the subject has completed baseline clinical assessments.

### Blinding

The present trial is a single-blinded trial. Outcome assessors and people responsible for the statistical analysis will be blinded to the randomization status.

### Data collection and management

Study-related information, such as participant’s identity, the data collected relating to the study, and medical records, will remain confidential. Case report forms (CRFs) will be completed on paper forms. Data will then be entered and stored in a password-protected electronic database. Original paper copies of CRFs and all study data will be stored in a locker with access allowed to the involved researchers only.

### Data analysis

The intention-to-treat (ITT) principle will be used in the statistical analysis. All participants randomized were involved in the ITT analysis. Data will be summarized using means (± SDs) for continuous variables. An analysis of covariance (ANCOVA) model will be used to compare the mean changes of outcomes from baseline to end of intervention, including baseline measurement as covariates. A paired *T* test or the Wilcoxon rank test will be used to compare variables from baseline to the end of intervention. Data will be presented as frequency for categorical variables. The chi-square test or Fisher’ exact test will be performed to examine differences. *P* < 0.05 will be considered statistically significant. All analysis will be performed using SAS version 9.3.

### Ethics and dissemination

The present study is registered at ClinicalTrials.gov (NCT02608554), and conducted in accordance with the Declaration of Helsinki. The study received approval from the First Affiliated Hospital of Heilongjiang University of Chinese Medicine Institutional Review Boards (approval number HZYLLKT201500201). The result of the present study will be published in a peer-reviewed journal.

### Safety

All adverse events reported during the study period will be documented in the CRF.

Serious adverse events (SAEs) will be notified to the principle investigator and within 24 h. All SAEs must be reported on the Adverse Event page of the CRF. The principal investigator is responsible for the management of the safety reporting according to local guidelines.

## Discussion

To the best of our knowledge, this is the first study to determine the effectiveness and feasibility of Tai Chi in treating PCOS. The result of the present study should provide evidence to assess the feasibility of a larger multicenter, randomized controlled trial in the future. One limitation is that the present study design did not include an assessment of the physiological benefits of Tai Chi. The Polycystic Ovary Syndrome Health-Related Quality of Life Questionnaire (PCOSQ) or other questionnaires will be employed in further study [23].

### Trial status

Trial start date: October 2016.

Currently recruiting (*N* = 37, January 2018).

This protocol version number is version 1.0.

## Additional file

Additional file 1: Standard Protocol Items: Recommendations for Interventional Trials (SPIRIT) 2013 Checklist: recommended items to address in a clinical trial protocol and related documents*. (DOC 131 kb)

## Abbreviations

ANCOVA: Analysis of covariance
AND: Androstadiendione
BMI: Body Mass Index
CHOL: Cholesterol
CRF: Case report form
CVD: Cardiovascular diseases
DHEAS: Dehydroepiandrosterone sulfate
E: Estradiol
FG: Ferriman-Gallwey
FSH: Follicle-stimulating hormone
GnRH: Gonadotropin-releasing hormone
GnRH: Gonadotropin-releasing hormone
HDL-C: High-density lipoprotein cholesterol
ICSI: Intracytoplasmic sperm injection
ITT: Intention-to-treat
IVF: In vitro fertilization
LDL-C: Low-density lipoprotein cholesterol
LH: Luteinizing hormone
OGTT: Oral glucose tolerance test
PCOS: Polycystic ovary syndrome
PCOSQ: The Polycystic Ovary Syndrome Health-Related Quality of Life Questionnaire
SAE: Serious adverse event
SHBG: Sex-hormone-binding globulin
SPIRIT: Standard Protocol Items: Recommendations for Interventional Trials
T: Testosterone
TG: Triglycerides
WHO: World Health Organization

## References

[1] Azziz R, Carmina E, Chen Z, Dunaif A, Laven JS, Legro RS, Lizneva D, Natterson-Horowtiz B, Teede HJ, Yildiz BO (2016). Polycystic ovary syndrome. Nat Rev Dis Primers.

[2] Ehrmann DA (2005). Polycystic ovary syndrome. N Engl J Med.

[3] Orio F, Palomba S (2014). Reproductive endocrinology: new guidelines for the diagnosis and treatment of PCOS. Nat Rev Endocrinol.

[4] World Health Organisation, International Association for the Study of Obesity, International Obesity Task Force (2000). The Asia-Pacific perspective: redefining obesity and its treatment.

[5] Barlow SE (2007). Expert committee recommendations regarding the prevention, assessment, and treatment of child and adolescent overweight and obesity: summary report. Pediatrics.

[6] Lim SS, Davies MJ, Norman RJ, Moran LJ (2012). Overweight, obesity and central obesity in women with polycystic ovary syndrome: a systematic review and meta-analysis. Hum Reprod Update.

[7] Bu Z, Dai W, Guo Y, Su Y, Zhai J, Sun Y (2013). Overweight and obesity adversely affect outcomes of assisted reproductive technologies in polycystic ovary syndrome patients. Int J Clin Exp Med.

[8] Huang K, Liao X, Dong X, Zhang H (2014). Effect of overweight/obesity on IVF-ET outcomes in Chinese patients with polycystic ovary syndrome. Int J Clin Exp Med.

[9] Goodman NF, Cobin RH, Futterweit W, Glueck JS, Legro RS, Carmina E (2015). American Association of Clinical Endocrinologists, American College of Endocrinology, and Androgen Excess and PCOS Society Disease State Clinical Review: guide to the best practices in the evaluation and treatment of polycystic ovary syndrome—part 1. Endocr Pract.

[10] Goodman NF, Cobin RH, Futterweit W, Glueck JS, Legro RS, Carmina E (2015). American Association of Clinical Endocrinologists, American College of Endocrinology, and Androgen Excess and PCOS Society Disease State Clinical Review: guide to the best practices in the evaluation and treatment of polycystic ovary syndrome—part 1. Endocr Pract.

[11] Gateva A, Unfer V, Kamenov Z (2018). The use of inositol(s) isomers in the management of polycystic ovary syndrome: a comprehensive review. Gynecol Endocrinol.

[12] Seyam E, Al Gelany S, Abd Al Ghaney A, Mohamed MAA, Youseff AM, Ibrahim EM, Khalifa EM, Hefzy E (2018). Evaluation of prolonged use of statins on the clinical and biochemical abnormalities and ovulation dysfunction in single young women with polycystic ovary syndrome. Gynecol Endocrinol.

[13] Chen Y, Wang XJ, Jin HL, Jin L (2013). Effects of resolving method of Chinese medicine on the lipid metabolism in polycystic ovary syndrome accompanied with non-alcoholic fatty liver disease. Zhongguo Zhong Xi Yi Jie He Za Zhi.

[14] Yang J, Liu Y, Huang J, Xu J, You X, Lin Q, Zhang J, Dun J, Huang S (2017). Acupuncture and Chinese medicine of artificial cycle therapy for insulin resistance of polycystic ovary syndrome with phlegm damp type and its mechanism. Zhongguo Zhen Jiu.

[15] Zhuo D (1982). Preventive geriatrics: an overview from traditional Chinese medicine. Am J Chin Med.

[16] Sun J, Buys N (2015). Community-based mind-body meditative Tai Chi program and its effects on improvement of blood pressure, weight, renal function, serum lipoprotein, and quality of life in Chinese adults with hypertension. Am J Cardiol.

[17] Hartley L, Flowers N, Lee MS, Ernst E, Rees K (2014). Tai chi for primary prevention of cardiovascular disease. Cochrane Database Syst Rev.

[18] Tsang T, Orr R, Lam P, Comino E, Singh MF (2008). Effects of tai chi on glucose homeostasis and insulin sensitivity in older adults with type 2 diabetes: a randomized double-blind sham-exercise-controlled trial. Age Ageing.

[19] Shayya R, Chang RJ (2010). Reproductive endocrinology of adolescent polycystic ovary syndrome. BJOG.

[20] Zhao X, Ni R, Li L, Mo Y, Huang J, Huang M (2011). Defining hirsutism in Chinese women: a cross-sectional study. FertilSteril.

[21] Hui SS, Xie YJ, Woo J, Kwok TC (2015). Effects of Tai Chi and walking exercises on weight loss, metabolic syndrome parameters, and bone mineral density: a cluster randomized controlled trial. Evid Based Complement Alternat Med.

[22] Johanson GA, Brooks GP (2010). Initial scale development: sample size for pilot studies. EducPsychol Meas.

[23] Lin CY, Ou HT, Wu MH, Chen PC (2016). Validation of Chinese version of Polycystic Ovary Syndrome Health-Related Quality of Life Questionnaire (Chi-PCOSQ). PLoS One.

